# *In vitro* Pharmacokinetics/Pharmacodynamics Evaluation of Fosfomycin Combined with Amikacin or Colistin against KPC2-Producing *Klebsiella pneumoniae*

**DOI:** 10.3389/fcimb.2017.00246

**Published:** 2017-06-16

**Authors:** Wei Yu, Kai Zhou, Lihua Guo, Jinru Ji, Tianshui Niu, Tingting Xiao, Ping Shen, Yonghong Xiao

**Affiliations:** ^1^State Key Laboratory for Diagnosis and Treatment of Infectious Disease, Collaborative Innovation Center for Diagnosis and Treatment of Infectious Diseases, The First Affiliated Hospital, College of Medicine, Zhejiang UniversityHangzhou, China; ^2^Department of Infectious Diseases, Zhejiang Provincial People's HospitalHangzhou, China

**Keywords:** pharmacokinetics/pharmacodynamics, fosfomycin, amikacin, colistin, KPC-producing *Klebsiella pneumoniae*

## Abstract

**Objectives:** The emergence of carbapenem-resistant *Enterobacteriaceae*, especially *Klebsiella pneumoniae*, has become a major concern in clinic settings. Combination therapy is gaining momentum to counter the secondary resistance and potential suboptimal efficacy of monotherapy. The aim of this study was to evaluate the bactericidal effect of fosfomycin (FM), amikacin (AMK), or colistin (COL) alone and combinations against KPC2-producing *K. pneumoniae* using dynamic model by simulating human pharmacokinetics *in vitro*.

**Methods:** The Pharmacokinetics Auto Simulation System 400 system was employed to simulate different dosing regimens of FM, AMK, and COL alone and combination. Bacterial growth recovery time (RT) and the area between the control growth and antibacterial killing curves (IE) were used as unbiased and comprehensive means for determining the antimicrobial effect.

**Results:** We observed that COL alone was much pronounced than FM or AMK against KPC-Kp. IE of FM (8 g every 8 h) plus AMK (15 mg/kg once-daily) and FM (8 g every 8 h) plus COL (75,000 IU/kg every 12 h) were higher (>170 and >200 LogCFU/mL·h^−1^, respectively) than that of monotherapies against sensitive strains. Of note, the rate of resistance was lower when using the combination of FM (8 g every 8 h) plus COL (75,000 IU/kg every 12 h) than using COL (75,000 IU/kg every 12 h) alone.

**Conclusions:** The combination of FM (8 g every 8 h) plus AMK (15 mg/kg once-daily) and FM (8 g every 8 h) plus COL (75,000 IU/kg every 12 h) were effective at maximizing bacterial killing and suppressing emergence of resistance.

## Introduction

In 2013, the Centers for Disease Control and Prevention declared that carbapenem-resistant *Enterobacteriaceae* (CRE) has become an urgent public health threat and required immediate and aggressive action (Centers for Disease Control and Prevention, 2013). Combination therapy is becoming a popular strategy to combat the emerging CRE, with the additional benefits of antimicrobial synergy and breadth of antimicrobial spectrum (Rafailidis and Falagas, 2014). Antimicrobial combination therapy has been widely used in clinical practice in a variety of clinical infections due to KPC-producing *Klebsiella pneumoniae* (KPC-Kp), however, robust evidence is lacking especially when β-lactam agents are not part of combination therapy regimen.

Fosfomycin (FM) and colistin (COL), as two recovering antibiotics, are considered to be the alternative treatments in clinical practice (Kanj and Kanafani, 2011). The main reason for both drugs revival was the inability of current antibiotics to confront these infections, characterized by high mortality rates and the paucity of new antimicrobial agents (Patel et al., 2008). In addition, it has been suggested that amikacin (AMK) is generally more active against extended-spectrum beta-lactamase (ESBL)-producing and quinolone-resistant *Escherichia coli* than other aminoglycosides, making it as a better option in cases of suspected infections caused by multidrug-resistant *Enterobacteriaceae* (Leibovici et al., 2009; Hanberger et al., 2013; NIH, 2016). Clinical evidence supporting FM combined with COL or AMK as a treatment option remains unclear. However, *in vitro* chequerboard synergistic test and time-kill assays have demonstrated that FM combined with AMK or COL could prevent the emergence of FM-resistant mutants and enhance bacterial killing against *Pseudomonas aeruginosa, K. pneumonia*, or *Acinetobacter baumannii* (Albur et al., 2015; Sime et al., 2016). In addition, our unpublished data also indicated that FM combined with COL or AMK had a significant additive effect against 136 KPC-Kp strains. Therefore, reliable pharmacokinetic (PK) and pharmacodynamic (PD) data are urgently needed to redefine appropriate dosing strategies for combinations so as to maximize clinical efficacy, reduce the developing rate of bacterial resistance, and minimize adverse effects. In this study, we simulated human exposures of FM, AMK, and COL alone and various combinations of these drugs *in vitro* against KPC-Kp infections, in order to verify the appropriate combination dosage regimen.

## Methods

### Bacterial strains

Three KPC-Kp strains were used in this study, including ATCC BAA-1705 and two clinical isolates (17186 and 18253) recovered from blood and urine, respectively. Carbapenemase-producing phenotype was detected using modified Hodge test, according to Clinical and Laboratory Standards Institute (CLSI) guidelines (Clinical and Laboratory Standards Institute, 2016). *Escherichia coli* ATCC 25922 and *K. pneumoniae* ATCC BAA-1705 and BAA-1706 were used as referring strains. Antimicrobial susceptibilities, molecular type and carbapenemase gene of *K. pneumoniae* ATCC BAA-1705, 17186, and 18253 are presented in Table 1. They were ST258, ST11, and ST690, respectively. The three strains were resistant to imipenem and ertapenem. ATCC BAA-1705 was resistant to AMK (MIC = 64 mg/l), while 17186 and 18253 were susceptible to AMK. In addition, 18253 was resistant to COL (MIC > 32 mg/l) but the other two strains were susceptible to COL.

**Table 1:** Characteristics of three KPC-producing *K. pneumoniae* strains in pharmacokinetics/pharmacodynamics study.

**Isolates**	**MIC**					**MLST**	**Carbapenemase genes**	**Specimen**
	**FM**	**IMP**	**ETP**	**COL**	**AMK**			
1705	1	4	8	0.5	64	258	bla_KPC-2_	ATCC
17186	8	16	128	0.25	1	11	bla_KPC-2_	Blood
18253	16	16	256	>32	4	11	bla_KPC-2_	Blood

*1705, ATCC BAA-1705; FM, fosfomycin; IMP, imipenem; ETP, ertapenem; COL, colistin; AMK, amikacin; MIC, minimum inhibitory concentration; FICIs, fractional inhibitory concentration indices*.

### Antibiotic dosing

The time-concentration data of FM, AMK, and COL after single intravenous administration was derived from previous studies (Lanao et al., 1981; Garraffo et al., 1990; Sauermann et al., 2005; Mizuyachi et al., 2011). The regimens administrated by intravenous (I.V.) infusion were simulated over 24 h: FM 8 g every 8 h (FM 8 g q8h) (Sauermann et al., 2005), AMK 7.5 mg/kg once-daily (AMK 7.5 mg/kg qd) (Lanao et al., 1981), AMK 15 mg/kg once-daily (AMK 15 mg/kg qd) (Garraffo et al., 1990), COL 75,000 IU/kg every 12 h (COL 75,000 IU/kg q12h) (Mizuyachi et al., 2011), FM (8 g q8h)/AMK (7.5 mg/kg qd), FM (8 g q8h)/AMK (7.5 mg/kg q12h), FM (8 g q8h)/AMK (15 mg/kg qd), FM (8 g q8h)/COL (75,000 IU/kg q12h) (The Supplemental Table 1 showed the pharmacokinetics parameters of different regimens and Supplemental Figure 2 showed the concentrations during the simulation).

### *In vitro* PK model and antibacterial activity measurement

The *in vitro* Pharmacokinetics Auto Simulation System 400 (PASS 400) used in the study was a product of DAINIPPON SEIKI (Kyoto, Japan) (Supplemental Figure 1). Briefly, the system consisted of central unit, fluid reservoir (containing fresh Mueller-Hinton broth and antibiotics) and waste unit. The three units were connected by sterile gas-tight syringe, filter and pipe. The central unit contained the same broth with only one kind of bacterial culture (control growth experiments) or bacterial culture plus antibiotic (killing and regrowth experiments) and was incubated at 37° in a shaking water bath. Magnetic rod was rotated in central unit to homogenize the whole system. A constant volume of the central unit controlled by PASS-402W software was requested from accurate simulations of the PK profiles. There were four syringe pump, including two drug pumps, one medium pump, and one waste fluid/sampling pump, constantly adjusted fluid volume. The drug syringe pumped for liquid transfer in the simulation of antibiotics alone or combination. Medium syringe pump was pumped into the culture vessel (central unit). Waste was pumped from the central unit for sampling tube. PK data were imputed in the software and then transferred to the control device, in order to accurately operate the pumps.

A two-compartment model was designed to expose bacteria to dynamic antibiotic concentrations by mimicking human PK. Growth curves in antibiotic-free simulations were as control. 0.5 McFarland standard cell densities were diluted for 100 times in the central compartment, ~10^6^ CFU/ml. Samples (1.5 ml) were collected at 0, 1, 2, 4, 6, 8, 10, 12, 14, 17, 20, 24 h and serially diluted in 0.9% sodium chloride before plating on MH agar plates. All plates were incubated overnight at 37°C. Only plates with 30–300 colonies were counted.

### PD analysis

The PD parameters included Maximum Kill Down (MKD), Maximum Kill Time (MKT), Area Above Kill Curve (AAKC), Bacterial growth recovery time (RT), -1Log Kill Time (−1KT), −2Log Kill Time (−2KT), −3Log Kill Time (−3KT), Regrowth Recovery Time (SRT), +1Log Growth Time (+1RT), Total Area Above Kill Curve (TAAKC), Analysis Start Time (AST), Total −1Log Kill Time (T-1KT), Total −2Log Kill Time (T-2KT), and Total −3Log Kill Time (T-3KT) (The Supplemental Figure 3 showed the analysis parameters). IE is defined as the area between the control growth and antibacterial killing curves determined to the end of the regrowth phase. RT and IE provide the most unbiased and comprehensive means for determining the antimicrobial effect (Firsov et al., 1997). Furthermore, IE is preferable to RT because IE, being an integral parameter, is determined not only by two points on the regrowth and normal growth curves but by all the points. IE was calculated by the trapezoidal rule using the program GraphPad Prism 5. Other parameters were calculated by PASS 400 Analyze Bactericidal Activity software.

### Emergence of resistance

The emergence of resistance to FM, AMK, and COL was assessed at 24 h of post-exposure. Samples were plated onto MHB agar plates without antibiotic or with antibiotics at 2 × and 4 × MIC. After overnight incubation, single colonies were selected to test MICs using two-fold serial agar dilution method again.

## Results

### Bacterial time-kill effect

**Figure 1:**
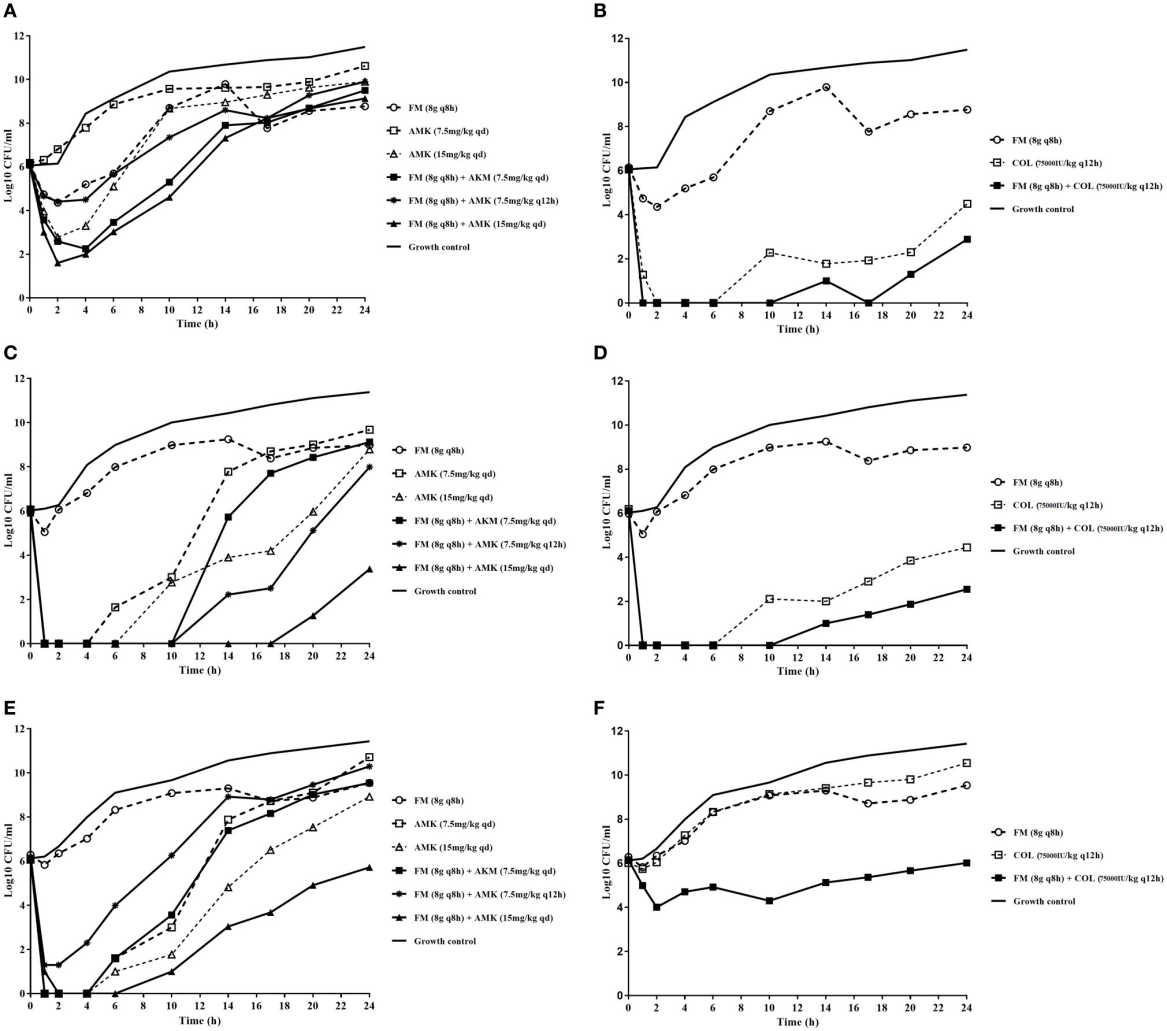
*In vitro* dynamic model time-kill assays using concentrations of fosfomycin, amikacin, and colistin (either alone or in combination) against three KPC-producing *K*. *pneumoniae* strains. **(A)** and **(B)** mono or combination therapy, respectively, against isolate ATCC BAA-1705; **(C)** and **(D)** mono or combination therapy, respectively, against isolate 17186; **(E)** and **(F)** mono- and combination therapy, respectively, against isolate 18253. FM, fosfomycin; AMK, amikacin; COL, colistin. The dotted lines indicate monotherapy, and the solid lines indicate combination therapy. Antibiotic concentrations are denoted by different symbols.

The dynamic time-kill experiment indicated that the combination of FM/AMK or FM/COL achieved an improved effect against KPC-Kp comparing to monotherapies, although re-growth was observed (Figure 1). AMK and COL alone showed more bactericidal effect than FM and suppressed the growth of KPC-Kp at the duration of the experiment. FM (8 g q8h) monotherapy had little discernible effect. However, for AMK monotherapy, increased dosage (from 7.5 mg/kg qd to 15 mg/kg qd) showed more bactericidal effects against susceptible strains 17186 and 18253 (Figures 1C,E).

In FM/AMK combination, once daily dosage regimen for AMK yielded more efficient antimicrobial effects than every 12 h administration. Furthermore, for FM/AMK combination against 18253, although initial bacterial killing at 6 h was similar to 17186, later bacterial killing was significantly lower than observed with the combination against 17186. However, no appropriate FM/AMK combination showed bactericidal activity against AMK-resistant strain ATCC BAA-1705 at 24 h (Figure 1A).

Notably, FM/COL also showed synergistic activity, despite the fact that isolate 18253 was COL resistant. It is noteworthy that ATCC BAA-1705 and 17186 were susceptible to COL, and that FM/COL showed bactericidal activity over a longer period of time with these two organisms. More rapid bacterial killing was observed for FM (8 g q8h)/COL (75,000 IU/kg q12h) combination against the KPC-Kp isolates (Figures 1B,D,F). FM/COL combination caused a >3 log10 reduction in CFU/ml at 24 h against COL susceptible strains compared to the initial count.

### PD parameters of different regimen against KPC-Kp

The PD parameters were analyzed by PASS 400 Analyze Bactericidal Activity software (Table 2). In the FM (8 g q8h) single-drug time-kill studies, RT (6.55 h) was longer against ATCC BAA-1705 than the other two strains, which were consistent with the sensitive status of those strains. For FM/AMK combination, the IE of FM (8 g q8h)/AMK (15 mg/kg qd) against ATCC BAA-1705, 17186 and 18253 were 96.7, 218.05 and 174.28 LogCFU/mL·h^−1^, respectively. Indeed, COL monotherapy showed greater effects against COL susceptible strains than FM and AMK monotherapies. The higher MKD (>6 LogCFU/mL), longer RT (>24 h), -3KT (>17 h), and larger IE (>100 LogCFU/mL·h^−1^) were observed for COL (75,000 IU/kg q12h). Interesting, for FM (8 g q8h)/COL (75,000 IU/kg q12h) combination, the IE was increased up to over 200 for ATCC BAA-1705 and 17186. For COL resistant isolate 18253, IE of FM/COL combination was still more than 100 LogCFU/mL·h^−1^ and SRT was above 20 h.

**Table 2:** Pharmacodynamics parameters of different regimen against KPC-producing *K. pneumoniae*.

**Dosage regimen**		**MKD (ΔLogCFU/mL)**	**MKT (h)**	**AAKC (ΔLogCFU/mL)**	**RT (h)**	**−1KT (h)**	**−2KT (h)**	**−3KT (h)**	**SRT (h)**	**+1RT (h)**	**T-1KT (h)**	**T-2KT (h)**	**T-3KT (h)**	**IE (LogCFU/mL•h-1)**
1705	FM (8 g q8h)	−1.8	1.9	6.9	6.6	3.2	NA	NA	4.7	7.4	NA	NA	NA	53.9
	AMK (7.5 mg/kg qd)	NA	0.0	0.0	NA	NA	NA	NA	0.0	NA	NA	NA	NA	17.0
	AMK (15 mg/kg qd)	−3.5	2.6	15.2	7.2	5.6	4.1	2.2	4.6	8.2	NA	NA	NA	55.1
	COL (75000 IU/kg q12h)	−6.3	7.2	>108.5	>24	>23.8	23.2	21.7	>16.8	NA	23.8	NA	NA	193.1
	FM (8 g q8h)/AMK (7.5 mg/kg qd)	−4.0	3.4	28.2	11.7	9.5	8.2	4.2	8.3	13.1	NA	NA	NA	87.8
	FM (8 g q8h)/AMK (7.5 mg/kg q12h)	−1.7	3.1	8.0	7.0	4.5	NA	NA	3.9	9.5	NA	NA	NA	58.8
	FM (8 g q8h)/AMK (15 mg/kg qd)	−4.7	2.6	32.7	12.2	10.5	8.4	5.2	9.7	13.6	NA	NA	NA	96.7
	FM (8 g q8h)/COL (75000 IU /kg q12h)	−6.6	1.4	>129.8	>24	>23.9	>23.7	>23.6	>22.6	NA	23.9	23.8	23.6	218.7
17186	FM (8 g q8h)	−0.9	1.0	1.1	1.9	NA	NA	NA	1.0	4.4	NA	NA	NA	35.1
	AMK (7.5 mg/kg qd)	−6.6	1.4	51.6	11.9	11.1	10.4	9.6	10.5	12.8	NA	NA	NA	108.0
	AMK (15 mg/kg qd)	−6.7	1.4	74.3	20.2	18.7	16.5	11.7	18.8	21.6	NA	NA	NA	153.4
	COL (75000 IU/kg q12h)	−6.7	1.4	>100.2	>24	>23.9	22.3	17.5	>22.6	NA	23.9	NA	NA	182.7
	FM (8 g q8h)/AMK (7.5 mg/kg qd)	−6.4	1.4	69.1	14.2	13.2	12.4	11.7	12.8	15.3	NA	NA	NA	138.4
	FM (8 g q8h)/AMK (7.5 mg/kg q12h)	−6.6	1.4	96.9	21.0	19.9	18.8	17.5	19.6	22.3	NA	NA	NA	180.1
	FM (8 g q8h)/AMK (15 mg/kg qd)	−6.6	1.4	>131.7	>24	>23.9	>23.8	22.4	>22.6	NA	23.9	23.8	NA	218.1
	FM (8 g q8h)/COL (75000 IU/kg q12h)	−6.6	1.4	>126.8	>24	>23.9	>23.7	>23.6	>22.6	NA	23.9	23.8	23.6	209.9
18253	FM (8 g q8h)	−0.5	1.0	0.5	1.9	NA	NA	NA	1.0	4.8	NA	NA	NA	30.0
	AMK (7.5 mg/kg qd)	−6.6	1.4	50.5	11.5	10.9	10.3	9.7	10.1	12.0	NA	NA	NA	105.8
	AMK (15 mg/kg qd)	−6.6	1.4	65.3	16.0	14.2	12.9	11.6	14.6	18.6	NA	NA	NA	139.0
	COL (75000 IU/kg q12h)	−0.3	1.0	0.3	2.0	NA	NA	NA	NA	1.0	3.6	NA	NA	21.4
	FM (8 g q8h)/AMK (7.5 mg/kg qd)	−6.6	1.4	51.3	12.2	11.1	10.2	9.3	10.9	13.5	NA	NA	NA	109.6
	FM (8 g q8h)/AMK (7.5 mg/kg q12h)	−5.2	1.4	25.9	9.8	7.7	5.8	4.5	8.4	10.9	NA	NA	NA	73.5
	FM (8 g q8h)/AMK (15 mg/kg qd)	−6.6	7.9	>93.5	>24	20.9	18.1	14.0	>16.1	NA	NA	NA	NA	174.3
	FM (8 g q8h)/COL (75000 IU/kg q12h)	−2.2	2.4	>27.1	>24	13.3	1.3	NA	>21.6	NA	NA	NA	NA	111.3

*NA, Not Applicable; MKD, Maximum Kill Down; MKT, Maximum Kill Time; AAKC, Area Above Kill Curve; RT, Bacterial growth recovery time; −1KT, −1Log Kill Time; −2KT, −2Log Kill Time; −3KT, −3Log Kill Time; SRT, Regrowth Recovery Time; +1RT, +1Log Growth Time; TAAKC, Total Area Above Kill Curve; T-1KT, Total −1Log Kill Time; T-2KT, Total −2Log Kill Time; T-3KT, Total −3Log Kill Time; IE, the area between the control growth and antibacterial killing curves; FM, fosfomycin; COL, colistin; AMK, amikacin*.

### Emergence of resistance

Antibiotic susceptibility test was performed again after 24 h in PASS 400 simulation studies. There was no growth on plates with 2 × MIC or 4 × MIC antibiotics at 24 h except for the cultures with FM (8 g q8h)/AMK (7.5 mg/kg q12h) and COL (75,000 IU/kg q12h) (Table 3). Isolates recovered from cultures with FM (8 g q8h)/AMK (7.5 mg/kg q12h) invariably showed increasing MICs of FM (MIC = 64 mg/l or 512 mg/l, respectively) and AMK (MIC > 512 mg/l). The MICs of COL monotherapy against COL—sensitive strains (ATCC BAA-1705 and 17186) were 32 mg/l and 8 mg/l, respectively. Interestingly, combination of FM/COL against COL—sensitive strains decreased the emergence of resistance to COL (MIC = 0.5 mg/l or 0.25 mg/l, respectively).

**Table 3:** Minimum inhibitory concentrations of fosfomycin, colistin, and amikacin against three KPC-producing *K. pneumoniae* strains after 24 h in pharmacokinetics simulation studies.

**Isolates**	**Dosage regimen**	**FM**	**AMK**	**COL**
ATCC-1705	FM (8 g q8h)	1	64	0.5
	AMK (7.5 mg/kg qd)	1	64	0.5
	AMK (15 mg/kg qd)	1	64	0.5
	COL (75000 IU/kg q12h)	1	64	32
	FM (8 g q8h)/AMK (7.5 mg/kg qd)	1	64	0.5
	FM (8 g q8h)/AMK (7.5 mg/kg q12h	512	>512	0.5
	FM (8 g q8h)/AMK (15 mg/kg qd)	1	64	0.5
	FM (8 g q8h)/COL (75000 IU/kg q12h)	1	64	0.5
17186	FM (8 g q8h)	8	1	0.25
	AMK (7.5 mg/kg qd)	8	2	0.25
	AMK (15 mg/kg qd)	8	1	0.25
	COL (75000 IU/kg q12h)	16	1	8
	FM (8 g q8h)/AMK (7.5 mg/kg qd	8	1	0.25
	FM (8 g q8h)/AMK (7.5 mg/kg q12h	64	>512	0.25
	FM (8 g q8h)/AMK (15 mg/kg qd)	8	1	0.25
	FM (8 g q8h)/COL (75000 IU/kg q12h)	8	1	0.25
18253	FM (8 g q8h)	16	4	>32
	AMK (7.5 mg/kg qd)	16	4	>32
	AMK (15 mg/kg qd)	16	4	>32
	COL (75000 IU/kg q12h)	16	4	>32
	FM (8 g q8h)/AMK (7.5 mg/kg qd	16	4	>32
	FM (8 g q8h)/AMK (7.5 mg/kg q12h	512	>512	>32
	FM (8 g q8h)/AMK (15 mg/kg qd)	16	4	>32
	FM (8 g q8h)/COL (75000 IU/kg q12h)	16	4	>32

*FM, fosfomycin; COL, colistin; AMK, amikacin*.

## Discussion

In this study, we evaluated the actions of FM, AMK, and COL alone and combination in a dynamic PK simulation, in order to measure their effects on bacterial killing and suppression of resistance. Our results suggested that the addition of FM to AMK or COL resulted in more prominent bactericidal effect against KPC-Kp, especially for FM (8 g q8h)/AMK (15 mg/kg qd) and FM (8 g q8h)/COL (75,000 IU/kg q12h). The combination achieved more sustained antibacterial effects compared to either of the monotherapies. FM monotherapy had little discernible effect, while higher dose and once daily dosage regimen of AMK showed more bactericidal effect against susceptible strains (Figures 1C,E). COL alone was more significantly bactericidal than FM and AMK. It is noteworthy that FM (8 g q8h)/AMK (7.5 mg/kg q12h) was more likely to induce drug resistance for the duration of the experiment, and the resistance was lower with the FM (8 g q8h)/COL (75,000 IU/kg q12h) combination regimen compared to COL (75,000 IU/kg q12h) monotherapy. Although, the exact molecular mechanisms are unknown, the PD interaction between FM and AMK may occur due to enhanced access for the AMK into target site within the bacterial ribosome. However, for FM and COL, both agents are bactericidal with different mechanisms of action on separate bacterial targets, with COL being active against the bacterial cell membrane and FM against the bacterial cell wall. Lahiri et al. (2015) described a novel mechanism underlying the synergistic antibacterial activity of FM/COL combination by FM caused accumulation of superoxides in the cells.

In addition to improvement of bacterial killing by FM/AMK, the current study showed the IE of FM (8 g q8h)/AMK (15 mg/kg qd) against sensitive strains were higher (>170 LogCFU/mL·h^−1^) than monotherapies and lower dose (Table 2). In a previous hollow-fiber infection model, consistent with our findings, the authors demonstrated that monotherapy with AMK but not FM was bactericidal, while FM/AMK combination was observed a rapid killing and growth of resistant strains were effectively suppressed (Sime et al., 2016). The promising therapeutic option of FM/AMK is currently being tested for clinical effectiveness (NIH, 2016). However, it should be noted that using the combination of FM (8 g q8h)/AMK (7.5 mg/kg q12h) to treat KPC-Kp can invariably lead to resistance. This may be because AMK exhibits bactericidal activity in a concentration-dependent manner and once-daily dosing also has the potential to decrease the incidence of nephrotoxicity and ototoxicity associated with the use of aminoglycosides (Craig et al., 1991). Studies on *Enterococcal* endocarditis showed that a large reduction of bacterial loads was observed when using multiple-dosing regimens with bactericidal activity for efficacy (Craig, 1998). Therefore, physicians should evaluate the efficacy and inconclusive results on the advantages or disadvantages of AMK multiple-dosing regimens in clinical practice.

COL, categorized as cationic polypeptide antibiotics, is considered as a last resort in the treatment of severe infections caused by multidrug-resistant Gram-negative organisms. At present, very limited PK data are available on COL used in critically ill patients. PD parameters used to accurately predict COL efficacy and their optimal values such as Cmax/MIC, AUC/MIC, and T > MIC have not yet been clearly defined in humans (Michalopoulos et al., 2011). In addition, there were concerns of probable nephrotoxicity with dose escalation. Therefore, combination therapy is suggested to be an alternative approach to overcome these drawbacks (Markou et al., 2008). In this study, we found that for the monotherapies of three drugs, COL showed the most efficiently antibacterial effect against KPC-Kp. However, COL (75,000 IU/kg q12h) dosage regimen induced resistance at 24 h. FM/COL combination against COL susceptible strains caused a >3 log10 reduction in CFU/ml at 24 h compared to the initial count and the IE was increased more than 200. Interestingly, FM (8 g q8h)/COL (75,000 IU/kg q12h) combination regimen can suppress the emergence of COL-resistant subpopulations. A previous study using *in vitro* time-kill studies also observed that combination of FM/COL showed synergy against NDM-producing *K. pneumoniae* (Albur et al., 2015). A subsequent clinical studies echoed these concerns for *P. aeruginosa* and *A. baumannii*. Apisarnthanarak et al. (Apisarnthanarak and Mundy, 2012) conducted a retrospective study involving 49 patients with hospital-acquired bacterial pneumonia and ventilator-associated bacterial pneumonia due to carbapenem-resistant *P. aeruginosa*, they suggested an equivalency of regimens that contained doripenem (1 g, 4 h infusion) plus FM vs. FM plus COL (5 mg/kg/day in two divided doses). Similar to preliminary study, COL vs. FM/COL for treatment of carbapenem-resistant *A. baumannii* infections (Sirijatuphat and Thamlikitkul, 2014), the patients who received combination therapy (a combination of intravenous COL plus intravenous FM sodium at a dosage of 4 g every 12 h) had more favorable microbiological response and clinical outcomes and lower mortality than those who received COL alone (a dosage of 5 mg of COL base activity/kg of body weight/day).

A better understanding of PK can maximize clinical efficacy and minimize adverse effects and rate of resistance, especially for the optimization of COL and AMK doses in different routes of administration. The findings of this study are positive and encouraging in terms of FM/COL and FM/AMK combination. More studies of FM/COL and FM/AMK combination therapy in the form of animal experiments and eventually clinical trials are urgently needed to test the efficacy, safety and clinical outcomes for refractory infections.

Our *in vitro* dynamic study is more representative of *in vivo* metabolism and transfer of antibiotics than chequerboard assay and fixed concentration time-kill experiments. However, it also has several limitations. Firstly, it fails to account for the host immune response, in the presence of which the exposure-response relationship may be different. Non-etheless, although the *in vitro* dynamic PK/PD study without immunoreaction in this study, FM/COL and FM/AMK also showed effective microbiological outcomes. Further investigation is needed to define the effect of immune system.

## Conclusions

In conclusion, we have shown that combination of FM/COL and FM/AMK substantially increased the bactericidal activity of monotherapies against KPC-Kp in an *in vitro* dynamic PK/PD model, especially for FM (8 g q8h)/AMK (15 mg/kg qd) and FM (8 g q8h)/COL (75,000 IU/kg q12h). Also, combination therapy and once-daily amikacin dosing suppressed the emergence of resistant subpopulations. These results should be supported in further animal infection models and even prospective randomized controlled clinical trials, consideration should also be given to increasing the dosing recommendations of AMK, provided that toxicity remains acceptable.

## Author contributions

YX and WY developed the concept and designed the experiments. WY, LG, JJ, and TN performed experiments. WY and TX performed statistical analysis. YX, KZ, and PS gave conceptual advice. WY and KZ wrote the paper. All authors discussed the results and implications and commented on the manuscript at all stages.

### Conflict of interest statement

The authors declare that the research was conducted in the absence of any commercial or financial relationships that could be construed as a potential conflict of interest.

## Supplementary material

Supplementary Figure 1 **Pharmacokinetics Auto Simulation System 400**.

Supplementary Figure 2 **The time-concentration of antibiotics in the Pharmacokinetics Auto Simulation System 400**.

Supplementary Figure 3 **The sketch of pharmacodynamic parameters**. MKD, Maximum Kill Down; MKT, Maximum Kill Time; AAKC, Area Above Kill Curve; RT, Bacterial growth recovery time; −1KT, −1Log Kill Time; −2KT, −2Log Kill Time; −3KT, −3Log Kill Time; SRT, Regrowth Recovery Time; +1RT, +1Log Growth Time; TAAKC, Total Area Above Kill Curve; AST, Analysis Start Time; T-1KT, Total-1Log Kill Time.

Supplementary Table 1 **Pharmacokinetics parameters of different regimens**.
