# Supplementary figures and images for: In vitro Pharmacokinetics/Pharmacodynamics Evaluation of Fosfomycin Combined with Amikacin or Colistin against KPC2-Producing Klebsiella pneumoniae
Source: Front Cell Infect Microbiol. 2017 Jun 16;7:246. doi: 10.3389/fcimb.2017.00246 (PMC5472793; doi:10.3389/fcimb.2017.00246)

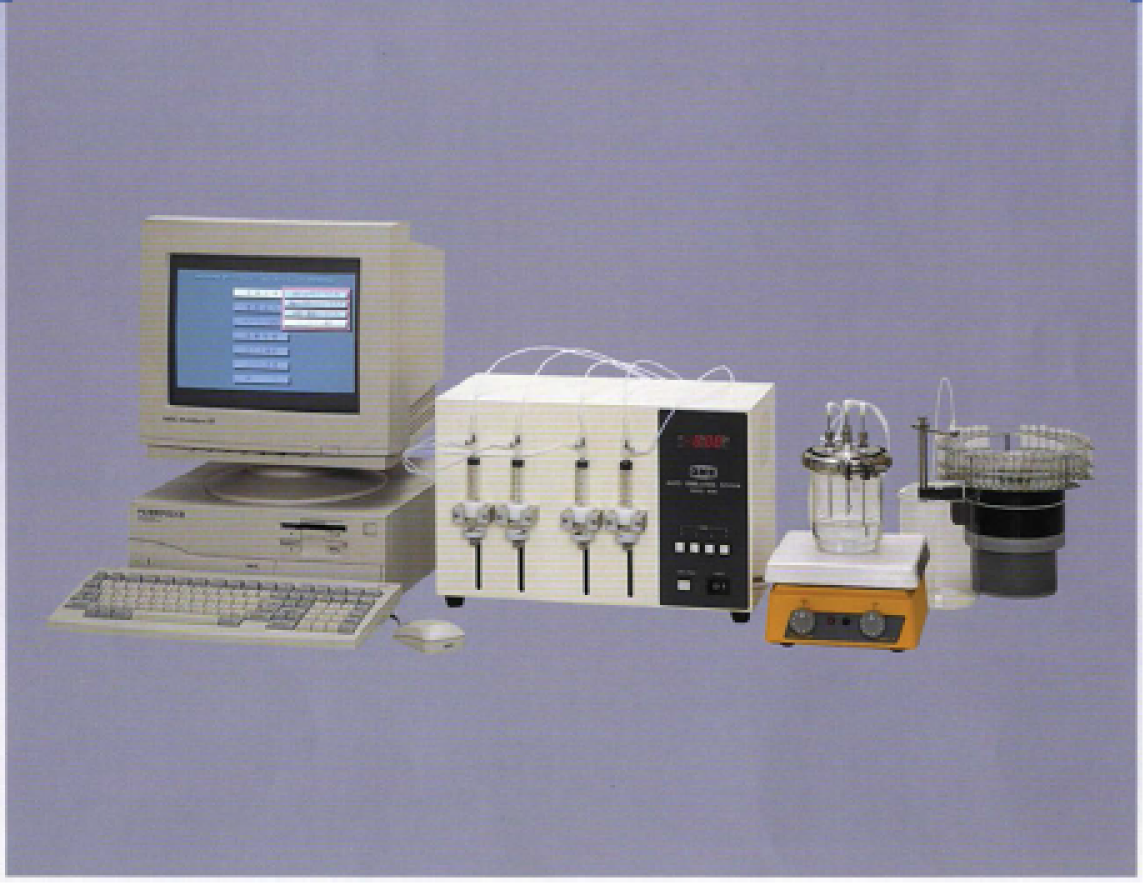

Supplement: Supplementary Figure 1 — Pharmacokinetics Auto Simulation System 400. [file Image1.TIF]

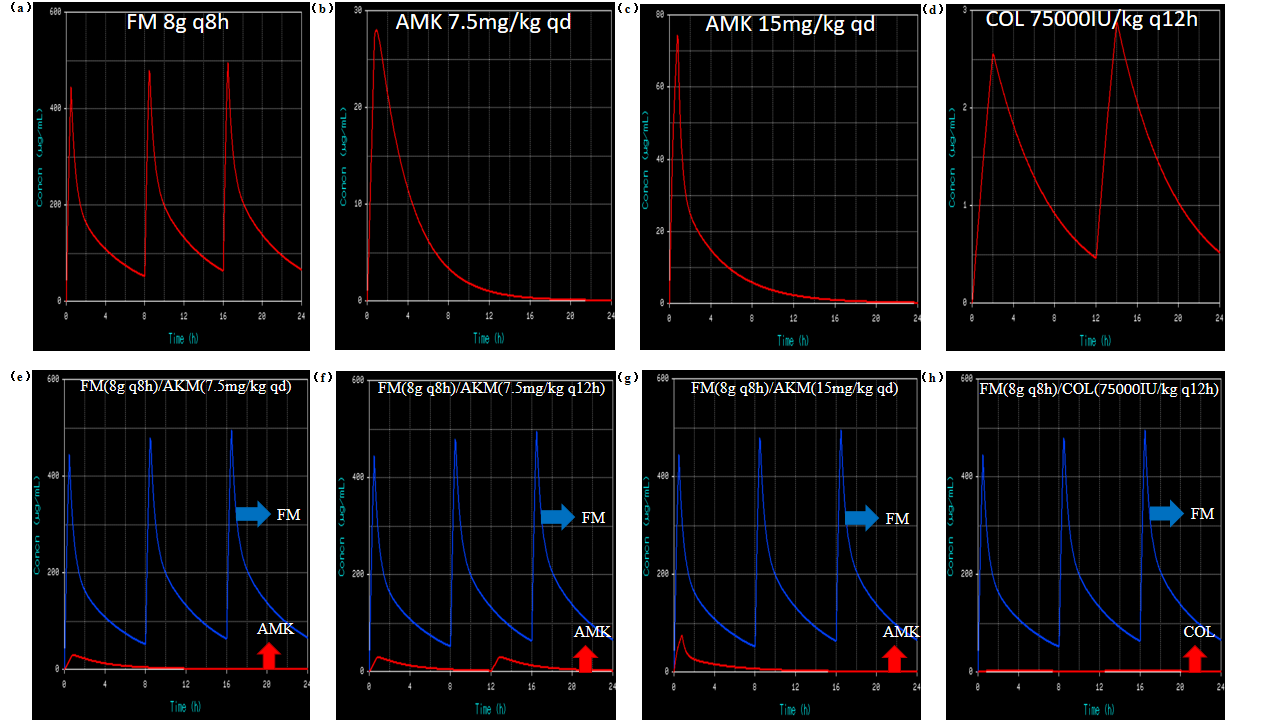

Supplement: Supplementary Figure 2 — The time-concentration of antibiotics in the Pharmacokinetics Auto Simulation System 400. [file Image2.TIF]

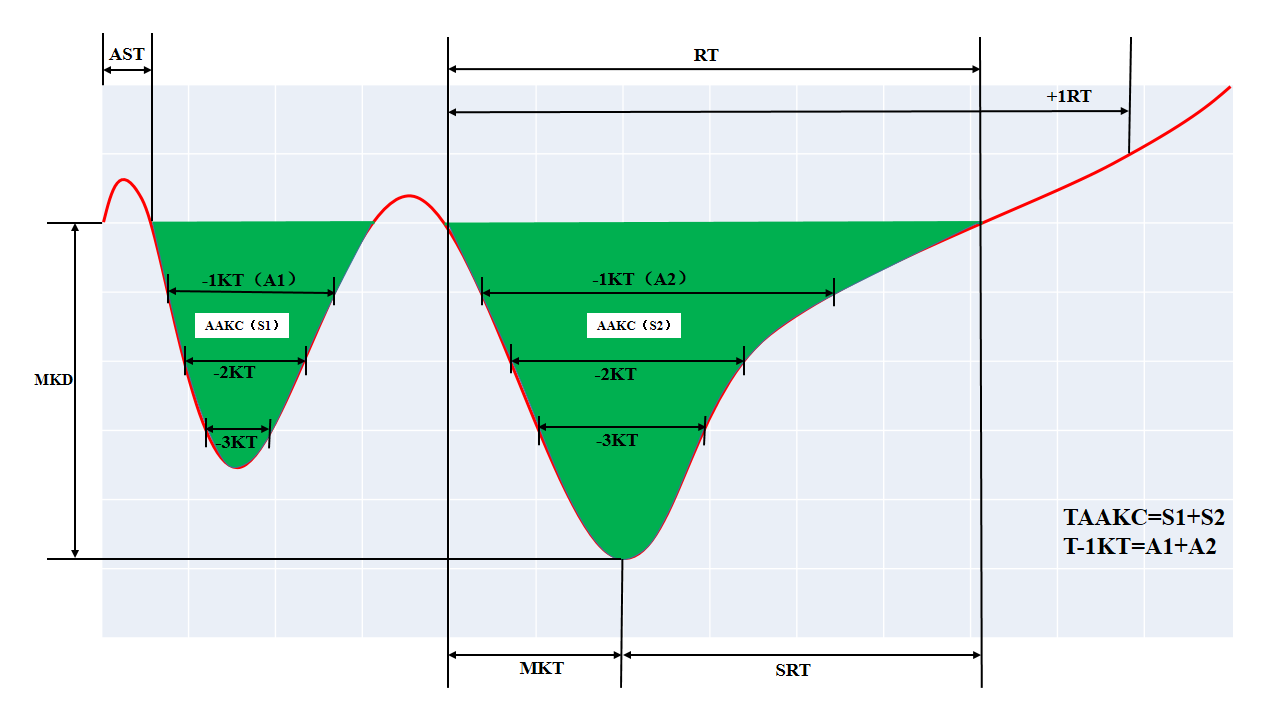

Supplement: Supplementary Figure 3 — The sketch of pharmacodynamic parameters. MKD, Maximum Kill Down; MKT, Maximum Kill Time; AAKC, Area Above Kill Curve; RT, Bacterial growth recovery time; −1KT, −1Log Kill Time; −2KT, −2Log Kill Time; −3KT, −3Log Kill Time; SRT, Regrowth Recovery Time; +1RT, +1Log Growth Time; TAAKC, Total Area Above Kill Curve; AST, Analysis Start Time; T-1KT, Total-1Log Kill Time. [file Image3.TIF]
