# Supplementary material for: In vitro Pharmacokinetics/Pharmacodynamics Evaluation of Fosfomycin Combined with Amikacin or Colistin against KPC2-Producing Klebsiella pneumoniae
Source: Front Cell Infect Microbiol. 2017 Jun 16;7:246. doi: 10.3389/fcimb.2017.00246 (PMC5472793; doi:10.3389/fcimb.2017.00246)
Supplement: Supplementary Table 1 — Pharmacokinetics parameters of different regimens. [file Table1.DOCX]

**Table 1.** Pharmacokinetics parameters of different regimens

| Regimens [11-14] | t1/2β(h) | AUC（mg·h/l) | CL（ml/min) | V(l) | Cmax(mg/l) |
| --- | --- | --- | --- | --- | --- |
| AMK 7.5 mg/kg once-daily | 2.229 ± 0.676 | 111.34 ± 28.643 | 74.178 ± 17.628 | 9.964 ± 4.493 | 27.98 |
| AMK 15 mg/kg once-daily | 2.37 ± 0.534 | 154.53 ± 29.91 | 112.833 ± 9.2 | 11.154 ± 1.776 | 76 |
| COL 75000 IU/kg every 12 hours | 4.00 ± 0.74 | 17.56 ± 6.80 | 10.525 ± 4.056 | 94.92 ± 30.193 | 2.55 |
| FM 8 g every 8 hours | 3.7 ± 2.2 | 1330 ± 609 | 126 ± 68 | 28.6 ± 9.9 | 443.64 |

FM, fosfomycin; COL, colistin; AMK, amikacin; t1/2β, elimination half-life; AUC, the area under the plasma concentration–time curve; ; CL, body clearance; V, volume of distribution; Cmax, maximum plasma concentrations; h, hour; l, liter.
