# Supplementary material for: In vitro Pharmacokinetics/Pharmacodynamics Evaluation of Fosfomycin Combined with Amikacin or Colistin against KPC2-Producing Klebsiella pneumoniae
Source: Front Cell Infect Microbiol. 2017 Jun 16;7:246. doi: 10.3389/fcimb.2017.00246 (PMC5472793; doi:10.3389/fcimb.2017.00246)
Supplement: Supplementary file 5 [file Presentation1.pdf]

Supplemental Figure 1. Pharmacokinetics Auto Simulation System 400.

Supplemental Figure 2. The time-concentration of antibiotics in the Pharmacokinetics Auto Simulation System 400.

Supplemental Figure 3. The sketch of pharmacodynamic parameters. MKD, Maximum Kill Down; MKT, Maximum Kill Time; AAKC, Area Above Kill Curve; RT, Bacterial growth recovery time; -1KT, -1Log Kill Time; -2KT, -2Log Kill Time; -3KT, -3Log Kill Time; SRT, Regrowth Recovery Time; +1RT, +1Log Growth Time; TAAKC, Total Area Above Kill Curve; AST, Analysis Start Time; T-1KT, Total -1Log Kill Time.
